# Evaluating 3D-Printed ABS and Carbon Fiber as Sustainable Alternatives to Steel in Concrete Structures

**DOI:** 10.3390/ma19020393

**Published:** 2026-01-19

**Authors:** Juan José Soto-Bernal, Ma. Rosario González-Mota, Judith Marlene Merida-Cabrera, Iliana Rosales-Candelas, José Ángel Ortiz-Lozano

**Affiliations:** 1 Department of Electronic Engineering, TecNM/Instituto Tecnologico de Aguascalientes, Av. Adolfo Lopez Mateos Ote. 1801, Bona Gens, Aguascalientes 20256, Mexico; j.j.soto.bernal@aguascalientes.tecnm.mx (J.J.S.-B.); iliana.rc@aguascalientes.tecnm.mx (I.R.-C.); 2 Department of Chemical and Biochemical Engineering, TecNM/Instituto Tecnologico de Aguascalientes, Av. Adolfo Lopez Mateos Ote. 1801, Bona Gens, Aguascalientes 20256, Mexico; rosario.gm@aguascalientes.tecnm.mx (M.R.G.-M.); j.marlene.merida@gmail.com (J.M.M.-C.); 3 Department of Civil Engineering, Universidad Autónoma de Aguascalientes, Av. Universidad 940 (Module 108), Ciudad Universitaria, Aguascalientes 20256, Mexico

**Keywords:** 3D printing, ABS reinforcement, CF reinforcement, compressive strength, anisotropic properties, concrete reinforcement, cement pastes, sustainability

## Abstract

This study evaluates the potential of 3D-printed acrylonitrile butadiene styrene (ABS) and carbon fiber (CF) as sustainable alternatives to steel reinforcement in cement-based materials. The experimental program analyzed the compressive strength of cement pastes and concrete cylinders incorporating 3D-printed ABS and CF elements. Unreinforced cement pastes exhibited higher compressive strength than reinforced pastes, indicating limited reinforcement–matrix interaction. In concrete cylinders, ABS reinforcement increased compressive strength by approximately 3 to 7 MPa compared to steel, whereas CF reinforcement showed variable performance and did not consistently surpass the control specimens. ANOVA and Tukey tests confirmed the statistical significance of the results. The anisotropic response of ABS and CF, inherent to layer-by-layer deposition, was identified as a major factor influencing structural performance, particularly with respect to reinforcement orientation. The results indicate that ABS presents potential as an environmentally favourable alternative to steel in selected applications, while CF requires further optimization for compression-oriented use. Continued research is recommended to evaluate long-term durability, environmental resistance, and reinforcement–matrix compatibility in order to advance the implementation of polymer-based, additively manufactured reinforcements in construction materials.

## 1. Introduction

Cement pastes, composed primarily of cement and water, serve as the foundation for many construction applications, including concrete and mortar mixtures [1,2]. The compressive strength of cement pastes is a critical characteristic that determines their ability to withstand loads without failure, while proper curing facilitates ongoing cement hydration, increasing strength over time. This compressive strength improves significantly during the early stages of curing [3]. Concrete, as a composite ceramic material, exhibits much higher compressive strength than tensile strength, largely due to hydration reactions that depend on time and the water-to-cement ratio (W/C) [1,3,4].

Standard test specimens for evaluating the compressive strength of concrete are typically cylinders with a diameter of 6″ (150 mm) and a height of 12″ (300 mm), as these dimensions ensure consistency and comparability in testing [4]. Other dimensions are sometimes used, provided that a length-to-diameter ratio of 2 is maintained [5].

Various approaches have been investigated to enhance the mechanical performance and durability of cement-based materials. Chemical modifications using nano-additives such as nano-SiO_2_, nano-TiO_2_, and nano-Al_2_O_3_ have demonstrated improvements in rheology, strength, and resistance to environmental degradation [6,7]. Other studies have evaluated the incorporation of different types of fibers, including steel and polypropylene fibers [8,9], dynamic-load-resistant fiber systems [10], natural fiber concrete with enhanced sustainability characteristics [11], and hybrid mixtures containing natural or recycled aggregates [12]. Additional research has explored non-conventional methods to modify early-age and hardened cementitious materials, including the application of static magnetic fields [13] and CO_2_ laser irradiation at different hydration stages [14]. Furthermore, the environmental burden associated with conventional steel reinforcement, particularly its high CO_2_ emissions during production [15], has encouraged the exploration of alternative reinforcement solutions based on materials with lower environmental impact.

The advent of 3D printing in construction has introduced a promising technology for creating complex, customized concrete structures with high precision while reducing material waste and construction time [16]. In addition, 3D printing enables the use of alternative materials, such as reinforced polymers, which have the potential to substitute steel in specific structural applications. Unlike steel, which exhibits isotropic properties with uniform mechanical behavior in all directions [17], 3D-printed materials such as acrylonitrile butadiene styrene (ABS) and carbon fiber (CF) are anisotropic. In these materials, mechanical strength varies with load direction due to the layer-by-layer deposition process inherent to 3D printing [18,19]. This anisotropy often results in higher strength along the X-Y plane (aligned with the layers) and reduced strength along the *Z*-axis (perpendicular to the layers) [20,21]. Such characteristics introduce unique considerations in structural applications, as layer orientation can significantly impact load distribution and resistance to compressive forces. This aspect is particularly relevant for reinforcement materials like CF, which, despite their stiffness, are more prone to microcracking when loads are applied perpendicular to the fiber direction [4].

The objective of this study is to evaluate the effectiveness of 3D-printed ABS and CF as sustainable reinforcement alternatives in concrete structures, focusing on their compressive strength, structural performance, and potential applications in construction. By assessing their impact on sustainability and exploring their feasibility for broader adoption, this research seeks to provide valuable insights into the viability of ABS and CF as reinforcement materials within the construction industry.

Methodologically, standard compressive strength tests were conducted using cylindrical specimens, as these are recognized for yielding consistent, comparable data. Additionally, an ANOVA test followed by a Tukey post hoc analysis was employed to determine the significance of differences between reinforcement types, chosen for its effectiveness in identifying specific group differences while controlling for Type I error. Such statistical rigor aims to support the study’s reliability and clarity in findings.

## 2. Materials and Methods

### 2.1. Materials

Experiments were conducted to compare the compressive strength of 150 mm height and 75 mm diameter cylinders made of cement paste and concrete [22,23,24] both unreinforced and reinforced with traditional steel elements and 3D-printed structures. The mechanical properties of reinforcement materials used in this work are summarized in Table 1.

The cement used in this study was a commercially available Portland cement classified as CEM I according to ASTM C150 and NMX-C-414-ONNCCE specifications. Its typical chemical composition, based on manufacturer data, consists predominantly of calcium oxide (CaO, 60–65%), silicon dioxide (SiO_2_, 18–22%), aluminium oxide (Al_2_O_3_, 4–7%), iron oxide (Fe_2_O_3_, 2–4%), magnesium oxide (MgO, 1–3%), and sulphates expressed as SO_3_ (2–3%). The representative particle size distribution of this cement ranges between 1 and 80 µm, with a median particle diameter (d_50_) of approximately 15–20 µm. These values are consistent with standard Portland cement manufactured for structural applications.

**Figure 1:**
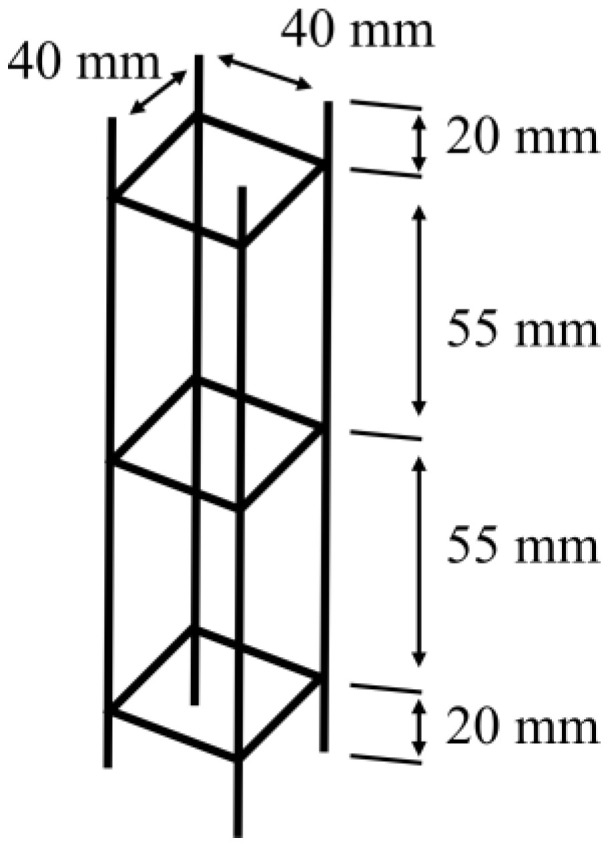
1:4 scale structure of the elements used.

For the reinforcement elements, a 1:4 scale was used for both metallic and 3D-printed elements, utilizing 1/16″ (1.6 mm) diameter rods divided into three chains, as shown in Figure 1 [5,22].

**Figure 2:**
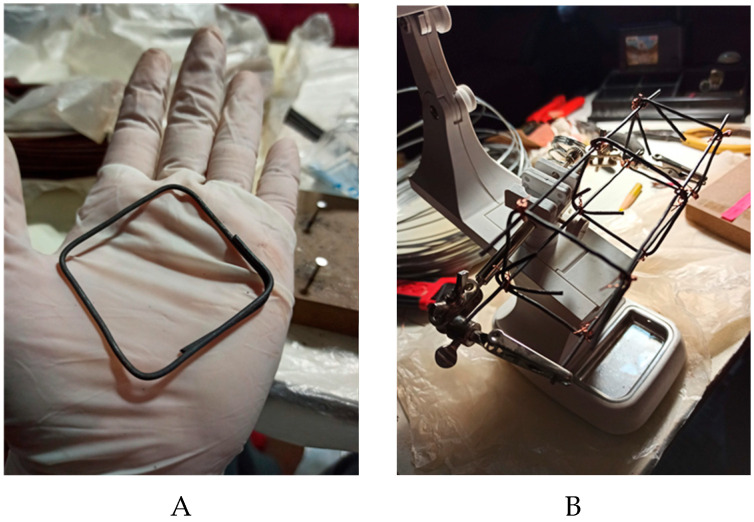

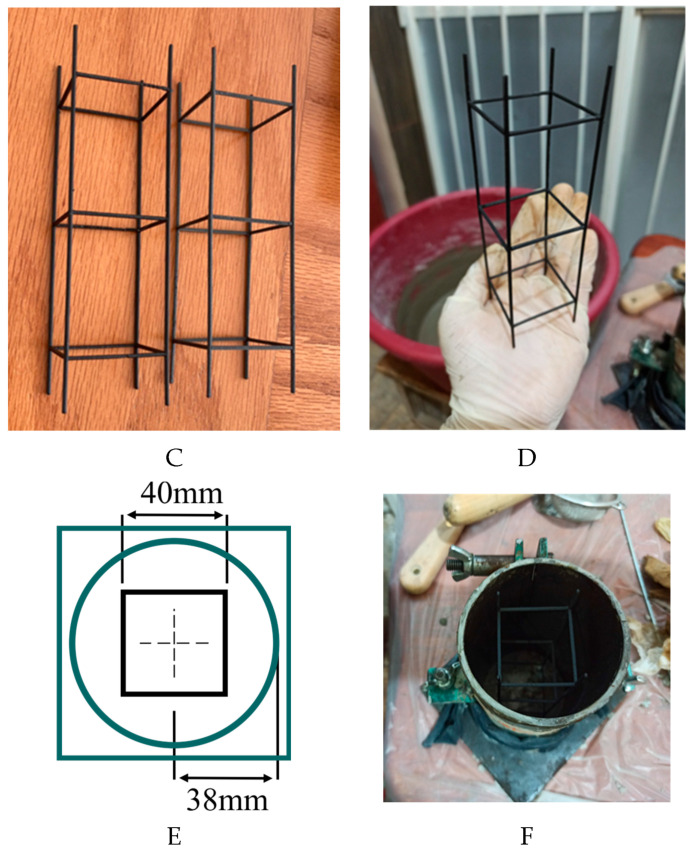
The 1:4 scale reinforcement elements used in the cylinders. (**A**): Metal stirrups; (**B**): Metal tie with 1/16″ rods; (**C**): 3D-printed CF and ABS elements; (**D**): View of an ABS element before placement in the cylinder; (**E**): Measurements for positioning the reinforcement elements; (**F**): Reinforcement element inside the steel mold.

The metallic elements were manually assembled (Figure 2A,B), and the 3D-printed elements were fabricated using carbon fiber (CF) filaments and acrylonitrile butadiene styrene (ABS) (Figure 2C,D), chosen for their mechanical properties and market availability [23,24]. Both 3D-printed and steel elements were centrally positioned in the cylinders (Figure 2E,F). Before casting the cement paste or concrete, the molds were coated with mineral oil to ensure that no reactions occurred between the mold and the concrete mix.

### 2.2. Filling the Steel Molds

**Figure 3:**
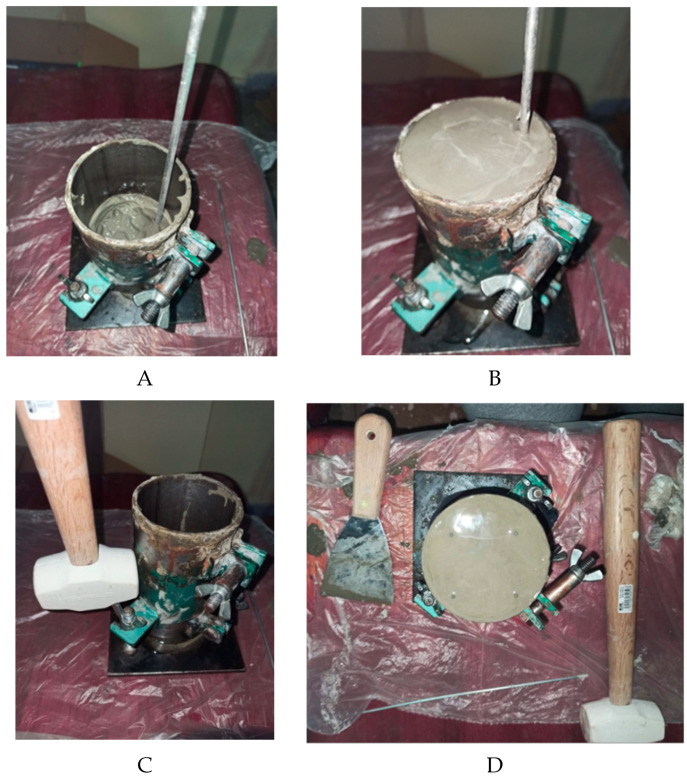
The process of filling the steel molds. (**A**): Rodding of the first layer; (**B**): Rodding of the final layer; (**C**): Tapping the molds with a rubber mallet; (**D**): Finished, compacted, and leveled mold with reinforcement element.

The 150 mm height and 75 mm diameter steel molds were filled using a scoop to ensure continuous mixing and prevent segregation [26]. A spatula was used along the upper edge of the mold while pouring the mix to ensure an even distribution. The filling was performed in three equal layers, compacting each layer by rodding it 25 times with a smooth 16 mm diameter, 600 mm long steel rod (Figure 3A,B) [27]. After compacting, the walls of the molds were tapped lightly with a rubber mallet (600 g ± 200 g, neoprene head and wooden handle) to eliminate as many air pockets as possible (Figure 3C) [26].

Care was taken to ensure that the final layer slightly overflowed after compaction. The surface was then leveled using a metal screed or trowel, ensuring a uniform and flat surface flush with the mold edges (Figure 3D) [5,26].

### 2.3. Preparation of Cement Pastes Families

Six families of cement pastes (M1-M6) were prepared. Each family consisted of four cylinders: one unreinforced, one with steel reinforcement, one with 3D-printed carbon fiber (CF), and one with 3D-printed ABS [22,23].

### 2.4. Preparation of Concrete Families

Similarly, six families of concrete cylinders (N1–N6) were prepared. Each family consisted of four cylinders: one unreinforced, one with steel reinforcement, one with 3D-printed carbon fiber (CF), and one with 3D-printed ABS. The concrete mixtures followed standard proportions for small-scale projects, consisting of 1.2 kg of cement, 2.47 kg of gravel, 2.05 kg of sand, and 0.78 kg of water, totaling 6.5 kg of concrete mix per sample. In this case, a concrete mix with a compressive strength of 15 MPa was used, which is the minimum strength value specified for structural concrete, according to [23].

### 2.5. Setting, Removal of Molds, and Curing

**Figure 4:**
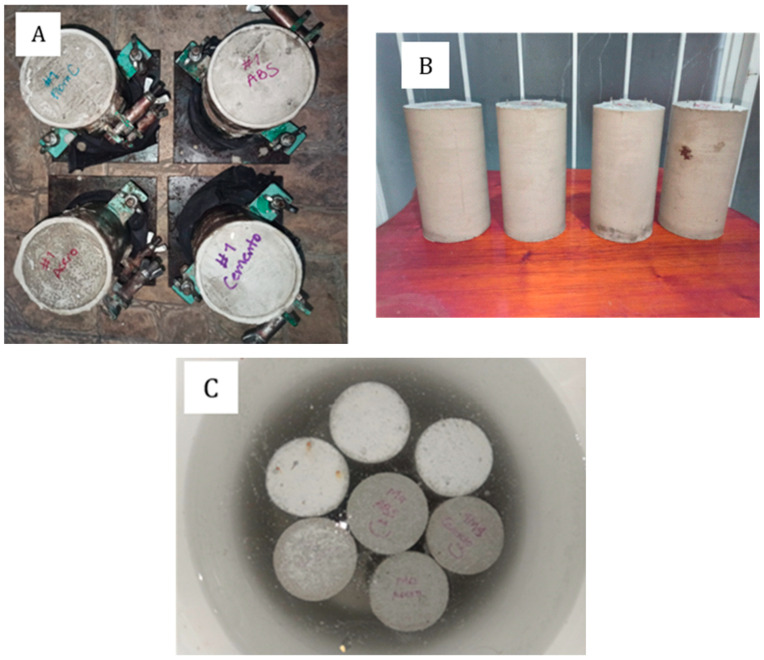
Setting, removal of molds, and curing. (**A**): Cylinders hardened in vertical position; (**B**): Cylinders removed from molds after 24 h; (**C**): Curing of the cement pastes.

After filling, the cylinders were placed on a rigid, flat, and horizontal surface free from vibrations. To prevent water evaporation, the molds were covered with wet burlap and sealed with a rubber membrane. They were stored at 23 °C and allowed to harden with the cylinder axis vertical (Figure 4A). Molds were removed after 24 h (Figure 4B).

After demolding, specimens were cured in water baths. The cylinders were placed in 20 L buckets containing 12 L of water saturated with 36 g of calcium hydroxide (Ca(OH)_2_, Sigma-Aldrich Corp., St. Louis, MO, USA) to prevent leaching [28]. The water temperature was maintained at 23 °C ± 2 °C, with the curing process lasting for 28 days (Figure 4C). During transportation to the testing facility, the cylinders were carefully wrapped in wet cloths to prevent moisture loss and minimize vibrations or impacts.

### 2.6. Testing

Compression tests were carried out at the Corporativo Construye S.A. de C.V. laboratory using the CR-2K compression testing machine (Sun Scientific Co., Ltd., Tokyo, Japan), certified by the Mexican Accreditation Entity (EMA) [29]. The compressive strength (*fc*) was calculated using Equation (1):

(1) $$f_{c}\;=\;\frac{F}{A}$$

where *fc* is the compressive strength (MPa), *F* is the maximum load (N), and *A* is the area of the specimen (mm^2^). According to standards, no correction for slenderness was required as the height was greater than 1.8 times the diameter of the cylinders [5].

The standard error of the mean (*SEM*) was calculated using Equation (2):

(2) $$SEM\;=\;\frac{s}{\sqrt{n}}$$

where *s* is the sample standard deviation and *n* is the number of specimens. The error bars in the plots represent ±*SEM* and are displayed above the mean values, since compressive strength cannot assume negative values.

### 2.7. Statistical Analysis

An analysis of variance (ANOVA) [30] was employed to identify significant differences among reinforcement types, followed by a Tukey post hoc test [31] to determine which specific groups differed from each other. This approach was chosen for its ability to handle multiple comparisons while controlling for Type I error, providing a robust framework for interpreting differences between sample groups.

## 3. Results

### 3.1. Compressive Strength of Cement Paste Cylinders

The compression test results for cement paste cylinders, both unreinforced and reinforced with steel, ABS, and CF, are shown in Table 2. The compressive strength was calculated using Equation (1), with a cross-sectional area (A) of 4477 mm^2^.

The compressive strengths calculated for these specimens are summarized in Table 3. Unreinforced cement paste demonstrated the highest average compressive strength (11.7 MPa), followed by CF-reinforced samples (10.1 MPa), ABS-reinforced samples (8.8 MPa), and steel-reinforced samples (8.1 MPa). The standard deviation (SD) values in Table 3 reflect the variability in strength measurements across samples, providing insight into the consistency of performance under compressive loading for each material. Unreinforced samples showed an SD of 2.35 MPa, while CF, ABS, and steel reinforcements had SDs of 1.87, 1.48, and 1.31 MPa, respectively.

### 3.2. Statistical Analysis of Cement Pastes

ANOVA was applied to identify statistically significant differences in compressive strength among the groups, followed by a Tukey post hoc test to determine specific pairwise differences. The analysis revealed a statistically significant reduction in compressive strength between unreinforced and steel-reinforced cement pastes, with a difference of −3.5 MPa (*p* = 0.0137). No statistically significant differences were observed between the unreinforced samples and those reinforced with ABS or CF (*p* > 0.05).

The standard deviation (SD) values indicate moderate variability in compressive strength across the different reinforcement materials, reflecting some consistency within each group. Furthermore, the standard error of the mean (SEM) values (CP = 0.96 MPa, CP with Steel = 0.54 MPa, CP with ABS = 0.60 MPa, and CP with CF = 0.76 MPa) quantify the precision of the average compressive strength measurements, highlighting slight variability within each group.

**Figure 5:**
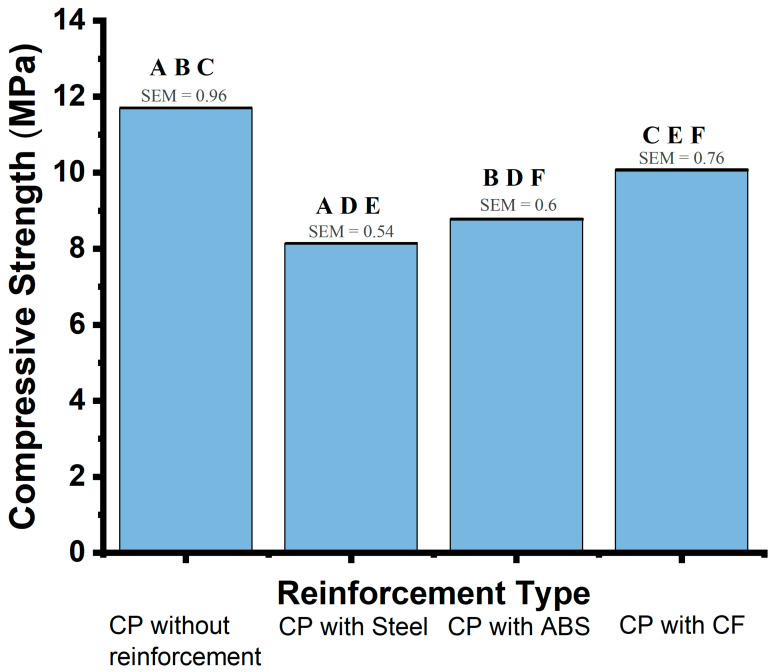
Compressive Strength Comparison for Cement Pastes (Tukey post hoc). Average compressive strength values (blue bars) with Standard Error of the Mean (SEM, red error bars) shown for each group. Letters above each bar indicate group comparisons using Tukey post hoc analysis.

Figure 5 shows the average compressive strength values for cement pastes (CP) with various reinforcement types: no reinforcement, steel, ABS, and CF. The standard error of the mean (SEM) is shown as error bars above each column, indicating the precision of the mean compressive strength measurements. Specific comparisons and significance levels are noted as follows:

**AA**: CP without reinforcement vs. Steel–A statistically significant reduction in compressive strength of −3.5 MPa (*p* = 0.014).

**BB**: CP without reinforcement vs. ABS–A reduction in strength of −2.9 MPa, with a trend close to significance (*p* = 0.0529).

**CC**: CP without reinforcement vs. CF–No statistically significant difference (−1.6 MPa, *p* = 0.443).

**DD**: ABS vs. Steel–No significant difference between ABS and steel, with a difference of 0.7 MPa (*p* = 0.9229).

**EE**: CF vs. Steel–CF displayed higher strength than steel, with a non-significant difference of 20.0 MPa (*p* = 0.2702).

**FF**: CF vs. ABS–CF showed higher strength compared to ABS, though not statistically significant (1.3 MPa, *p* = 0.604).

The results underscore the limited impact of reinforcement materials on compressive strength in cement pastes, with only steel reinforcement showing a statistically significant reduction in strength compared to the unreinforced paste.

**AA**: CP without reinforcement vs. Steel (significant reduction of −3.5 MPa, *p* = 0.014);

**BB**: CP without reinforcement vs. ABS (reduction of −2.9 MPa, trend close to significance, *p* = 0.0529);

**CC**: CP without reinforcement vs. CF (no significant difference, −1.6 MPa, *p* = 0.443);

**DD**: ABS vs. Steel (no significant difference, 0.7 MPa, *p* = 0.9229);

**EE**: CF vs. Steel (CF higher by 2.0 MPa, not significant, *p* = 0.2702);

**FF**: CF vs. ABS (CF higher by 1.3 MPa, not significant, *p* = 0.604).

### 3.3. Compressive Strength of Concrete Cylinders

The compression test results for the concrete cylinders, both unreinforced and reinforced with steel, ABS, and CF, are shown in Table 4. The compressive strength was calculated using Equation (1), with a cross-sectional area (A) of 4477 mm^2^.

The calculated compressive strengths for these specimens are further detailed in Table 5. Among the tested groups, ABS-reinforced concrete achieved the highest average compressive strength (24.0 MPa), followed by steel-reinforced concrete (22.0 MPa), and unreinforced concrete (19.0 MPa). CF-reinforced concrete exhibited the lowest average compressive strength (16.4 MPa).

The standard deviation (SD) values—1.18 MPa for ABS, 1.41 MPa for steel, 1.09 MPa for unreinforced concrete, and 1.01 MPa for CF—provide insight into the consistency of each material’s performance under compressive loading. The relatively low SD values across all groups suggest a consistent response to compressive loads within each reinforcement type, though slight variability was observed, reflecting material-specific behavior.

### 3.4. Statistical Analysis of Concrete Cylinders

The ANOVA test confirmed statistically significant differences among reinforcement types in concrete cylinders (*p* < 0.001). A subsequent Tukey post hoc analysis provided specific pairwise comparisons, revealing the following significant differences in compressive strength: ABS vs. unreinforced concrete, with an increase of 5.0 MPa (*p* < 0.001); steel vs. unreinforced concrete, with an increase of 3.0 MPa (*p* = 0.0017); and CF vs. unreinforced concrete, with a decrease of 2.6 MPa (*p* = 0.0058).

The standard deviation (SD) values (1.09 MPa for unreinforced concrete, 1.01 MPa for steel, 1.41 MPa for ABS, and 1.19 MPa for CF) highlight moderate variability in compressive strength measurements across reinforcement types, suggesting that ABS and steel provide more consistent compressive strength improvements than CF, which shows more limited effectiveness under compression.

The standard error of the mean (SEM) values further quantifies the precision of these measurements for each group: 1.09 MPa for unreinforced concrete, 1.01 MPa for steel, 1.41 MPa for ABS, and 1.19 MPa for CF. These SEM values indicate that, while ABS reinforcement yields the highest average compressive strength, it also displays slightly greater variability across samples compared to the other reinforcements.

**Figure 6:**
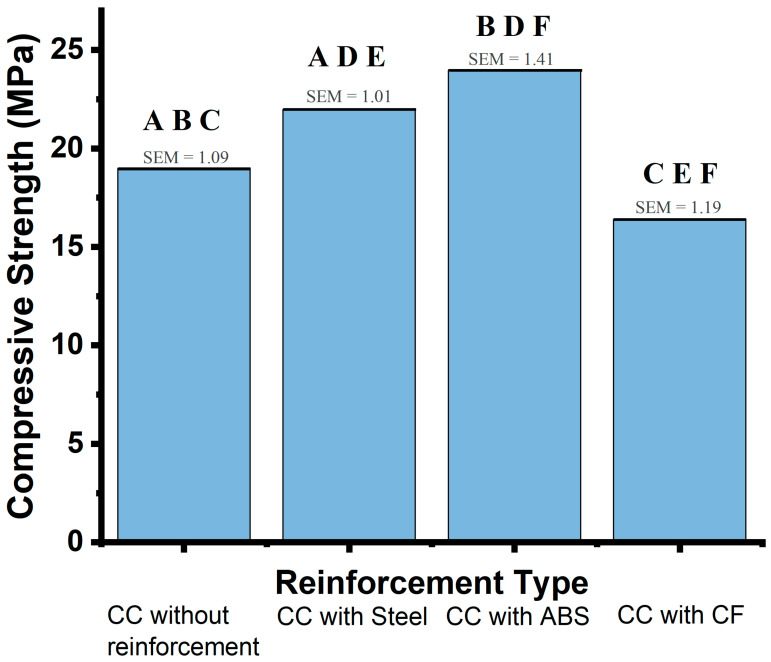
Compressive Strength Comparison for Concrete Cylinders (Tukey post hoc Analysis).

Figure 6 presents average compressive strength values (blue) and SEM (red) for concrete cylinders with various reinforcement types. The Tukey post hoc analysis results are represented by letter groupings above each bar:

**AA**: Steel vs. Concrete. An increase in strength (3.0 MPa, *p* = 0.0017).

**BB**: ABS vs. Concrete. A significant increase in compressive strength (5.0 MPa, *p* < 0.001).

**CC**: CF vs. Concrete. A significant reduction in strength (−2.6 MPa; *p* = 0.0058)

**DD**: ABS vs. Steel. ABS outperformed steel, with a difference of −2.0 MPa (*p* = 0.0369).

**EE**: CF vs. Steel. CF showed significantly lower strength compared to steel, with a difference of −7.6 MPa (*p* < 0.001).

**FF**: CF vs. ABS. CF showed significantly lower strength compared to ABS, with a difference of −7.6 MPa (*p* < 0.001).

Average compressive strength values (blue bars) are shown for concrete cylinders (CC) with different types of reinforcement: no reinforcement, steel, ABS, and CF. Standard Error of the Mean (SEM, red section) is displayed above each bar, providing a measure of precision in the mean compressive strength. Letter groupings above the bars indicate pairwise comparisons and significance levels identified in the Tukey post hoc analysis: 

**AA**: CC without reinforcement vs. CC with Steel (significant increase of 3.0 MPa, *p* = 0.0017);

**BB**: CC without reinforcement vs. CC with ABS (significant increase of 5.0 MPa, *p* < 0.001);

**CC**: CC without reinforcement vs. CC with CF (significant reduction of −2.6 MPa, *p* = 0.0058);

**DD**: CC with ABS vs. CC with Steel (ABS higher by 2.0 MPa, *p* = 0.0369);

**EE**: CC with CF vs. CC with Steel (CF significantly lower by −7.6 MPa, *p* < 0.001);

**FF**: CC with CF vs. CC with ABS (CF significantly lower by −7.6 MPa, *p* < 0.001).

## 4. Discussion

### 4.1. Anisotropy in 3D-Printed Reinforcements

The anisotropic nature of 3D-printed materials, such as ABS and carbon fiber (CF), significantly influences their mechanical performance in structural applications. Due to the layer-by-layer deposition process, these materials exhibit varying strengths depending on the load direction. The X-Y plane, which aligns with the layers, typically demonstrates higher strength and stiffness compared to the *Z*-axis, where interlayer adhesion is weaker. While this anisotropy benefits load distribution along the stronger plane, it may restrict compressive performance when loads are applied perpendicular to the layers, especially in CF, which is prone to buckling or early microcracking under such conditions.

### 4.2. Impact of Reinforcement on Compressive Strength

The results demonstrate that unreinforced cement pastes exhibited the highest compressive strength compared to reinforced pastes, possibly due to the homogeneity of the paste, which facilitates uniform stress distribution. In contrast, the addition of reinforcements can create stress concentration points that contribute to microcracks and ultimately reduce strength [32]. In reinforced samples, the interaction between the cement matrix and the reinforcement material significantly impacts overall performance. Inadequate adhesion and lack of aggregates may further reduce compressive strength in these materials [2].

In concrete samples, the inclusion of aggregates and the optimized interaction with reinforcement materials yielded significant improvements in compressive strength, particularly with ABS and steel reinforcements. The standard deviation values provided valuable insights into the consistency of each reinforcement’s performance. For instance, ABS-reinforced concrete displayed a standard deviation of 1.18 MPa, indicating a relatively stable performance across samples. Steel-reinforced concrete showed slightly greater variability with a standard deviation of 1.41 MPa, reflecting moderate variation in load response. Although CF-reinforced concrete exhibited lower compressive strength than unreinforced concrete, it displayed a low standard deviation (1.01 MPa), indicating consistent but limited performance. This consistency could be advantageous in applications with moderate load requirements, where predictability is valued over maximum load-bearing capacity.

The inherent anisotropy of 3D-printed reinforcements, such as ABS and CF, introduces additional considerations. The layer-by-layer construction of these materials creates interlayer interfaces that may act as points of weakness under compressive stress. In CF, this effect is pronounced due to its stiffness and brittleness, which can lead to microcracking or delamination along the layer boundaries. While ABS’s flexibility aids in distributing loads more evenly, the weaker bonds between layers may still limit its compressive performance when loads are not aligned with the X-Y plane. This anisotropic behavior underscores the importance of aligning load orientations with the material’s stronger axis in applications where 3D-printed reinforcements are used.

### 4.3. ABS Performance

The results of this study demonstrate that 3D-printed ABS elements can significantly enhance the compressive strength of concrete, outperforming both traditional steel reinforcement and unreinforced concrete. The ABS-reinforced concrete cylinders achieved a compressive strength of 24.0 MPa, surpassing both steel-reinforced samples (22.0 MPa) and unreinforced concrete (19.0 MPa). This superior performance highlights the potential of ABS as a viable alternative to steel in environments where corrosion resistance, reduced weight, and sustainability are essential.

The anisotropic properties of 3D-printed ABS play a critical role in its compressive performance. ABS demonstrates enhanced load distribution and resilience when loads align with the X-Y plane, where interlayer bonds are strongest. Despite the inherent variability that anisotropy can introduce, the fact that ABS outperforms steel in this study suggests it could be particularly advantageous in applications where load orientations are controlled or aligned with its strongest axis. Additionally, the flexibility of ABS in the X-Y plane allows for more uniform load distribution, which may provide benefits in specific applications compared to more rigid materials like steel. This characteristic of ABS highlights its adaptability and potential in structural applications requiring strategic load alignment.

### 4.4. CF Performance

The CF-reinforced concrete samples exhibited the lowest average compressive strength (16.4 MPa), even lower than that of the unreinforced concrete. This unexpected outcome aligns with previous research indicating that CF is more effective in tensile and flexural applications rather than in compression. The limited compressive performance of CF-reinforced concrete may stem from inadequate load transfer due to suboptimal bonding between the fibers and the concrete matrix. Additionally, the inherent stiffness and brittleness of CF tend to create stress concentration points, which can lead to early microcracking or buckling, thereby diminishing its effectiveness under compressive loads.

The CF-reinforced concrete samples showed a low standard deviation (1.01 MPa), suggesting consistent compressive performance across samples, despite the limited strength. While this predictability may be advantageous in applications with moderate or consistent loading requirements, the brittleness of CF and its susceptibility to early microcracking render it less suitable for applications where high compressive strength is critical. These findings indicate that the primary advantages of CF lie in tensile and flexural applications, while further optimization is required to enhance its performance in compression-based applications.

### 4.5. Statistical Analysis and Practical Implications

The ANOVA and Tukey post hoc tests confirmed statistically significant differences among the various reinforcement groups, with ABS exhibiting the highest overall increase in compressive strength relative to unreinforced concrete (*p* < 0.001). The practical implications of these findings are noteworthy. The incorporation of ABS in concrete reinforcement offers multiple benefits, including enhanced mechanical performance, reduced weight, and improved resistance to environmental degradation. Additionally, ABS is more cost-effective to produce compared to steel and is recyclable, aligning well with the increasing demand for sustainable construction materials.

The isotropic properties of steel provide a consistent mechanical response under multidirectional loads, which is advantageous in applications with variable or unpredictable load paths. This uniformity in strength makes steel a reliable choice in structural designs and methodologies that require stability across diverse loading scenarios. In contrast, the anisotropic nature of 3D-printed ABS and CF implies that their mechanical strength is influenced by load orientation, making them particularly effective in applications where load paths can be controlled to align with their stronger planes. However, this anisotropy may limit the use of ABS and CF in structural designs that demand uniform strength across all directions, potentially restricting their application in certain load-intensive environments.

These findings underscore the potential of ABS as a versatile reinforcement material in sustainable construction, particularly when load orientation can be managed, while also highlighting the need to carefully consider the directional dependency of 3D-printed materials like ABS and CF in structural applications.

### 4.6. Sustainability and Future Prospects

This research highlights the potential of ABS as a sustainable alternative to steel. ABS not only provides superior compressive strength but also offers environmental advantages, such as a significantly lower carbon footprint compared to steel. While steel production generates approximately 1.85 tons of CO_2_ per ton, contributing substantially to global emissions, the production of ABS emits only 2–4 kg of CO_2_ per kilogram, depending on energy sources and processes. Additionally, the inherent resistance of ABS to corrosion eliminates the need for additional protective treatments, thereby reducing both maintenance costs and environmental impact. From an economic perspective, typical 2024 market values indicate that conventional reinforcing steel ranges between 0.9 and 1.2 USD per kilogram, whereas commercial ABS filaments generally range between 1.5 and 2.0 USD per kilogram, and carbon fiber composite filaments may range between 18 and 25 USD per kilogram depending on formulation and manufacturer. Although carbon fiber remains significantly more expensive than steel, ABS presents only a moderate increase in cost, which may be compensated by its corrosion-free behavior and its suitability for modular, lightweight structural applications.

Future research should examine the long-term durability of ABS-reinforced concrete under varying environmental conditions, including exposure to moisture, temperature fluctuations, and chemical agents. Advances in multi-material 3D printing could further optimize reinforcement performance by allowing for customized layer orientations that address specific structural demands. Such improvements could fully leverage the anisotropic properties of ABS and CF, expanding their effectiveness as sustainable alternatives to traditional reinforcements in the construction industry.

### 4.7. Limitations

This study presents several limitations. First, the mechanical evaluation was restricted to compressive strength; tensile and flexural properties were not assessed, despite their structural relevance. Second, no microstructural or interfacial bond analyses were conducted, limiting insight into reinforcement–matrix interaction. Third, the mechanical properties of the 3D-printed ABS and CF elements were not experimentally validated but obtained from external references, which may not reflect printing-induced variability. Fourth, failure modes observed during compressive testing were not documented or analyzed. Fifth, the study does not include a cost–benefit assessment or discussion of regulatory compliance, limiting its immediate applicability. Additionally, the anisotropic nature of 3D-printed ABS and CF was not characterized through parameters such as Poisson ratio, tensile response, or lateral deformation under load. These properties are necessary for establishing a complete mechanical profile of the printed reinforcements and will be addressed in future work through dedicated tensile and flexural tests, as well as numerical modeling. These constraints should be addressed to strengthen future investigations.

## 5. Conclusions

This study demonstrates the feasibility of replacing steel elements in concrete structures with 3D-printed reinforcements, particularly ABS, which showed superior compressive strength compared to both steel and unreinforced concrete. The main findings can be summarized as follows:

1. Compressive Strength: ABS-reinforced concrete achieved an average compressive strength of 24.0 MPa, exceeding steel-reinforced concrete (22.0 MPa) and unreinforced concrete (19.0 MPa). These results indicate that ABS may serve as an effective reinforcement material in applications where compressive strength governs structural behavior.
2. Sustainability: ABS offers environmental advantages due to its lower carbon footprint, recyclability, and resistance to corrosion. These attributes may reduce long-term maintenance needs and support the adoption of more sustainable construction practices.
3. CF-reinforced concrete exhibited an average compressive strength of 16.4 MPa, which was lower than that of unreinforced concrete. This suggests that CF is more suited for applications where tensile behavior is critical, and further optimization is required for compression-oriented uses.
4. Structural Design Considerations: The results indicate that 3D-printed ABS has the potential to challenge steel in specific compressive applications; however, steel remains indispensable in situations requiring high tensile capacity and where established design methodologies provide proven reliability.
5. Anisotropy and Material Orientation: The anisotropic behavior of 3D-printed ABS and CF underscores the importance of aligning reinforcement orientation with dominant load paths. Optimized layer orientation and multi-material printing strategies could enhance load distribution and structural integrity.

Overall, this study highlights 3D-printed ABS as a promising reinforcement material, particularly in applications where its compressive strength, sustainability, and corrosion resistance provide measurable advantages. The findings support further research on advanced reinforcement geometries, multi-material printing, and anisotropy-driven design strategies in order to broaden the structural applications of polymer-based, additively manufactured reinforcements.

## 6. Future Work

Although the performance of ABS is very promising, further investigation is essential to understand its long-term behavior under varying environmental conditions, such as exposure to moisture, freeze–thaw cycles, and chemical agents. Additionally, its performance under lateral loads, its Young’s modulus, and resistance to repeated loading require in-depth study to ensure suitability across diverse structural applications. Exploring multi-material 3D printing technologies could enable the development of hybrid reinforcement structures, combining the strengths of multiple materials and potentially enhancing both compressive and tensile performance.

For CF, future research should focus on improving its compressive performance through approaches such as surface treatments to enhance bonding, as well as optimizing the orientation and configuration of fibers within the concrete matrix. Given the directional dependence of CF due to anisotropy, further testing under varied loading conditions, including lateral and cyclic loads, would provide a more comprehensive understanding of the mechanical behavior and potential structural applications of CF.

The anisotropic characteristics inherent in 3D-printed materials like ABS and CF, resulting from their layer-by-layer fabrication process, also merit further investigation. Specifically, the effects of layer orientation and interlayer bonding on load-bearing capacity should be studied in order to tailor reinforcement placement to specific stress demands and optimize structural integrity.

Future research may also explore mineral-filled polymer composites, including talc-reinforced ABS formulations, which have demonstrated potential for improving stiffness and dimensional stability in additive manufacturing. The integration of such composite materials could offer additional mechanical benefits while maintaining the corrosion-free behaviour and lightweight characteristics of polymer-based reinforcement systems.

Future work should also include tensile, flexural, and interlayer-bonding tests of the 3D-printed ABS and CF reinforcement elements in order to establish a complete mechanical comparison with conventional steel reinforcement.

Future investigations should also incorporate numerical modeling approaches, including finite element and computational fluid dynamics simulations, in order to evaluate stress distribution, reinforcement–matrix interaction, and the consistency between theoretical predictions and experimental responses.

Standardizing manufacturing and implementation processes will be essential for facilitating the widespread adoption of 3D-printed reinforcements in the construction industry. Ensuring that the lab-tested performance of these materials can be reliably replicated in real-world applications is key to maintaining structural safety and durability, supporting the broader acceptance of ABS, CF, and similar materials as viable alternatives to traditional reinforcements in structural design.

## Figures and Tables

**Table 1:** Mechanical properties of reinforcement materials used in concrete: Steel [17], 3D-Printed ABS [19,20], and 3D-Printed carbon fiber (CF) [21,25].

Property	Steel	3D-Printed ABS	3D-Printed Carbon Fiber (CF)
Tensile Strength (MPa)	400–550	30–50	61.3
Compressive Strength (MPa)	250–350	24	44.1
Young’s Modulus (GPa)	200	2–2.5	38.7
Density (g/cm3)	7.85	1.04	41.7
Anisotropy	Isotropic	Anisotropic (stronger along X-Y plane)	Anisotropic (stronger along X-Y plane)

**Table 2:** Compression test results (kN) for cement paste cylinders (CP) without reinforcement, with steel reinforcement, and with 3D-printed ABS and CF reinforcement.

Specimen	M1	M2	M3	M4	M5	M6
CP unreinforced	69.2	47.1	61.3	41.7	45.6	49.1
CP with Steel	32.9	41.2	44.1	30.4	30.9	39.2
CP with ABS	52.0	34.8	38.7	40.2	33.4	37.3
CP with CF	48.6	55.9	41.7	49.5	31.4	44.1

**Table 3:** Calculated compressive strength (MPa) for cement paste cylinders (CP) without reinforcement, with steel reinforcement, and with 3D-printed ABS and CF reinforcement.

Specimen	M1	M2	M3	M4	M5	M6	Average	Standard Deviation
CP unreinforced	15.4	10.5	13.7	9.3	10.2	11.0	11.7	2.35
CP with Steel	7.3	9.2	9.9	6.8	6.9	8.8	8.1	1.31
CP with ABS	11.6	7.8	8.7	9.0	7.4	8.3	8.8	1.48
CP with CF	10.8	12.5	9.3	11.1	7.0	9.9	10.1	1.87

**Table 4:** Compression test results (kN) for concrete cylinders (CC) without reinforcement, with steel reinforcement, and with 3D-printed ABS and CF reinforcement.

Specimen	N1	N2	N3	N4	N5	N6
CC unreinforced	85.8	87.1	75.0	86.4	88.3	87.3
CC with Steel	94.2	105.0	95.7	99.1	94.2	102.4
CC with ABS	101.5	115.3	99.1	112.8	109.9	105.9
CC with CF	81.9	67.7	72.6	71.1	77.5	69.7

**Table 5:** Calculated compressive strength (MPa) of concrete cylinders (CC) without reinforcement, with steel reinforcement, and with 3D-printed ABS and CF reinforcement.

Specimen	N1	N2	N3	N4	N5	N6	Average	Standard Deviation
CC unreinforced	19.2	19.5	16.8	19.3	19.7	19.5	19.0	1.09
CC with Steel	21.0	23.4	21.4	22.1	21.0	22.9	22.0	1.41
CC with ABS	22.7	25.7	22.1	25.2	24.5	23.7	24.0	1.18
CC with CF	18.3	15.1	16.2	15.9	17.3	15.6	16.4	1.01

## Data Availability

The original contributions presented in this study are included in the article material. Further inquiries can be directed to the corresponding author.
